# Mg(OH)_2_ Nanoflakes for Effective Removal of Phosphate Ions

**DOI:** 10.1002/open.70188

**Published:** 2026-04-17

**Authors:** Moses D. Ashie, Nicholas Chance, Sushmita Roy, Niroj Aryal, Bishnu Prasad Bastakoti

**Affiliations:** ^1^ Department of Chemistry North Carolina A&T State University Greensboro North Carolina USA; ^2^ Department of Natural Resources and Environmental Design North Carolina A&T State University Greensboro North Carolina USA

**Keywords:** adsorption, environmental remediation, phosphate ion, water quality, zeta potential

## Abstract

Simple, one‐pot, and low‐cost fabricated Mg(OH)_2_ nanoflakes, synthesized at room temperature, exhibited enhanced properties necessary for phosphate ion adsorption from solutions. Scanning electron microscopy and transmission electron microscopy showed flake‐like nanoparticles that were highly segregated, offering high surface area and active sites. Other characterization techniques, such as energy‐dispersive X‐ray analysis, X‐ray diffraction, and X‐ray photoelectron spectroscopy, contributed to the understanding of the chemical composition of the materials. The investigation of zeta potential revealed the surface charge of the nanoflakes before and after adsorption, supporting the adsorption mechanism of phosphate. Results from the ion adsorption experiment using ion chromatography confirmed that our fabricated material performed well in the phosphate adsorption test.

## Graphical abstract

The direct synthesis of Mg(OH)_2_ nanoflakes yielded a simple, low‐cost adsorbent for phosphate removal. Microscopy confirmed highly segregated flake‐like structures with large surface area and active sites. Zeta potential measurements showed changes in surface charge after adsorption, supporting the interaction mechanism between phosphate ions and Mg(OH)_2_ surfaces.

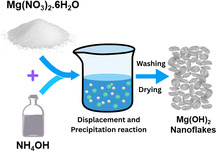

## 1 Introduction

Phosphorus, as phosphate (PO_4_^3−^), is a vital nutrient for all living creatures. Due to its biological significance and chemical versatility, phosphate is widely utilized in industrial processes, detergents, and agricultural fertilizers. Although phosphate is necessary for terrestrial ecosystems, high levels in aquatic systems can be toxic [1] and disrupt the delicate balance of water bodies. When large amounts of phosphate are introduced, the excessive presence of phosphorus in natural freshwater bodies can disturb the nutrient balance and lead to excessive growth of algae and aquatic plants [2]. Fish and other aquatic life have been reported to die due to eutrophication, a process caused by nutrient enrichment, primarily nitrogen and phosphorus, which also leads to unchecked algae growth, dissolved oxygen depletion, and disturbance of aquatic ecosystems. The usage of fertilizers and the release of untreated wastewater keep increasing phosphate concentrations in surface and groundwaters [3, 4]. Legislatively, phosphate concentrations in natural waters are expected to range from 0.01 to 0.1 mg/L. However, research has found that many water bodies close to urban and agricultural areas have concentrations higher than 1 mg/L [5]. The U.S. Environmental Protection Agency has set a limit of 0.025 mg/L for the phosphate limit [6] for reservoirs. Measures are therefore being put in place through research to prevent eutrophication [7, 8].

Chemical precipitation (using metal salts such as ferric chloride or aluminum sulfate), biological removal (utilizing enhanced biological phosphorus removal), and membrane filtration are examples of conventional phosphate removal methods. Despite their effectiveness, these methods frequently have limitations, including the production of sludge, operational complexity, high expenses, and inefficiency at low concentrations. For example, chemical precipitation produces significant amounts of sludge that require additional treatment and disposal, necessitating accurate dosage [9, 10, 11]. The adsorption‐based technologies, especially those that use nanomaterials, have drawn more interest for the removal of phosphate due to their high surface‐area‐to‐volume ratio, which significantly improves their ability to interact with the contaminants [12].

Among these numerous adsorbents, magnesium hydroxide [Mg(OH)_2_] has been particularly targeted as a promising adsorbent for phosphate removal [13]. It possesses good physicochemical properties that are conducive to adsorption, is abundant, affordable, and environmentally safe. The Mg(OH)_2_ structure enables surface complexation and strong electrostatic interactions with phosphate ions, particularly at acidic and neutral pH. It can also be used repeatedly in real‐world water treatment systems due to its high sorption capacity and outstanding regeneration potential. Its moderate alkalinity and buffering ability reduce the likelihood of large pH swings in treated water. This is important because Mg(OH)_2_ makes the treated water safer for human consumption and environmental discharge by removing toxic contaminants. In previous studies, pollutants such as antibiotics and heavy metals have been successfully removed using magnesium hydroxide nanoparticles [14]. Falyouna et al. showed that Mg(OH)_2_ was effective in eliminating antibiotics from aqueous medium [15], whereas Guo et al. reported results of the removal of Co (II) using magnesium hydroxide powder [16]. The primary mechanism of magnesium hydroxide‐assisted phosphate removal is surface adsorption, in which phosphate ions bind to hydroxyl groups on the Mg(OH)_2_ surface. Recent research has investigated the use of magnesium hydroxide nanowires for the specific purpose of phosphate removal [17, 18].

The main objective of this study is to synthesize, characterize, and evaluate two‐dimensional Mg(OH)_2_ nanoparticles for the removal of phosphate ions from aqueous solutions. We aimed to understand adsorption behavior and optimize process settings to achieve optimal removal efficiency by examining the effects of the initial phosphate concentration and solution pH. We fabricated Mg(OH)_2_ nanoflakes via precipitation, an economical, scalable, and environmentally safe synthesis technique. The nanoflakes’ morphology and other material properties were examined using scanning electron microscopy and transmission electron microscopy (SEM and TEM), X‐ray diffraction (XRD), X‐ray photoelectron spectroscopy (XPS), and a zeta analyzer. Even though surfactants are structure directing agents and improve morphology, these effective plate‐like materials were formed without the use of surfactants [19, 20, 21, 22]. The effectiveness of phosphate removal in water treatment applications was investigated and elucidated using zeta potential and adsorption tests. These findings support ongoing initiatives to protect aquatic ecosystems, combat eutrophication, and ensure the sustainability of the world's water supplies.

## 2 Experimental Section

### 2.1 Materials

Magnesium nitrate hexahydrate [Mg(NO_3_)_2_·6H_2_O] 99% was purchased from Sigma‐Aldrich. Ammonium hydroxide (NH_4_OH) 28%–30% was purchased from Thermo Scientific. Potassium nitrate (KNO_3_) 99% was purchased from Sigma‐Aldrich. Sodium hydroxide (NaOH), potassium hydrogen phosphate (KH_2_PO_4_), and hydrochloric acid (HCl) were purchased from Fischer Chemical. Distilled water was used in the synthesis of the samples. All chemicals used were analytical grade and were not further purified.

### 2.2 Synthesis of Magnesium Hydroxide Nanoflakes

Mg(OH)_2_ nanoflakes were synthesized using the precipitation method. To achieve this, 1 g of Mg(NO_3_)_2_·6H_2_O was dissolved in 50 mL of water under magnetic stirring. Four milliliters of NH_4_OH was added dropwise to the solution, which was stirred for 30 min. A cloudy solution was obtained. After mixing, the resulting solution was kept at room temperature for 2 h. The supernatant was carefully discarded by pipetting to avoid loss of precipitate due to the particles’ light nature. The remaining solution containing the precipitate was transferred to centrifuge tubes and centrifuged for 5 min at 4500 rpm. The precipitate obtained was washed three times with 18 MΩ‐cm water and dried in an oven at 60°C for 72 h. The dried Mg(OH)_2_ was then ground into a fine powder for further characterization.

### 2.3 Characterization of Magnesium Hydroxide Nanoflakes

To determine the physical and chemical properties of the fabricated materials, samples were analyzed using various analytical instruments and techniques. Fourier transform infrared spectroscopy (FTIR) by Shimadzu (IRTracer‐100 model) with a DLATGS detector, recording FTIR spectra from 400–4000 cm^−1^, helped identify the formed material. Images obtained from a field‐emission scanning electron microscope (FESEM, Model JSM‐IT800) provided valuable insights into the sample's morphology. Transmission electron microscopy (TEM, Model JEOL JEM‐2100plus) was used to examine the high‐resolution structure, and energy‐dispersive X‐ray (EDX) mapping revealed the elemental composition of the NPs. Elemental and chemical composition were examined using an XPS (Escalab Xi+). The zeta potential was measured using a Malvern Zetasizer to assess the surface charges of materials in solution. Information on crystallinity was obtained using a Rigaku Miniflex 600 X‐ray diffractometer, with spectra recorded from 10° to 90° using CuKα radiation (*k* = 1.5417 Å). The XRD analysis was conducted with a step size of 0.02°, a scan rate of 2°/min, a current of 15 mA, and an operational voltage of 40 kV.

### 2.4 Adsorption Experiments

Isotherm adsorption experiments were conducted to determine the adsorption capacities of Mg(OH)_2_ nanoflakes for phosphates. Phosphate solutions were made using KH_2_PO_4_. The pH of the working solution was adjusted with 0.1 M HCl and 0.1 M NaOH. For adsorption experiments, 20 mg of Mg(OH)_2_ nanoflakes was added to 50 mL of phosphate solution at concentrations ranging from 5 to 50 ppm, in increments of 5 ppm. After the addition of nanoflakes, the solutions were stirred for 30 min using the IKA C‐MAG MS 7 at a speed level of 2 and then centrifuged at 4500 rpm for 5 min. Subsequently, 2 mL of the supernatant was transferred to ion chromatography (IC) vials. All experiments were done in triplicate. Dionex 6000 IC was used for all quantifications. The method used a 2 × 250 mm analytical AS11‐HC‐4 µm column and AG11‐HC‐4 µm guard column, an eluent generator concentration of 35 mM KOH, an isocratic eluent flow rate of 0.25 ml/min, and a conductivity detector. The amount of adsorbate adsorbed per unit mass of adsorbent was calculated using the formula:

(1) $$q_{e}=\frac{(C_{o}-C_{e})\times V}{m}$$

where

*q*_*e*_ = amount of phosphate adsorbed

*C*_*o*_ = initial concentration (mg/L)

*C*_*e*_ = equilibrium concentration (mg/L)

*V* = volume of solution (L)

*m* = mass of adsorbent (g)

The isotherm was fitted using the equation:

(2) $$q_{e}=\frac{q_{m}K_{L}C_{e}}{1+K_{L}C_{e}}$$

where *q*_*m*_ is the maximum adsorption capacity and *K*_*L*_ is the Langmuir constant.

## 3 Results and Discussion

**FIGURE 1:**
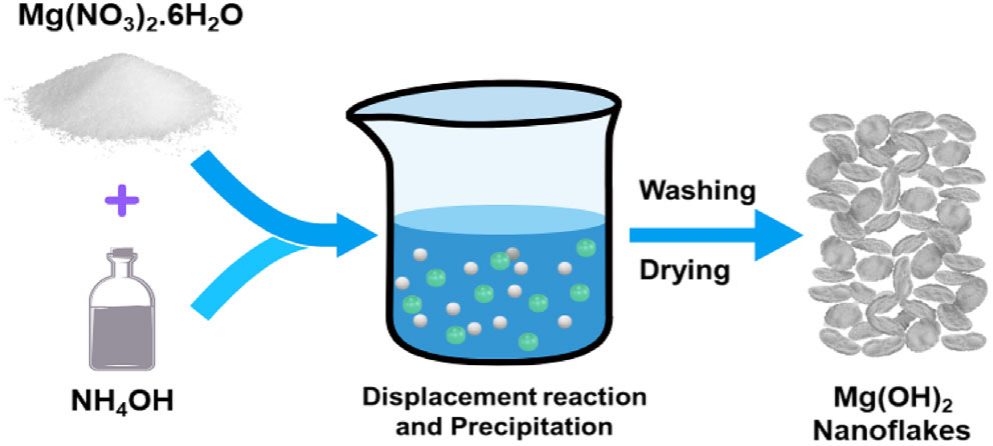
Schematic diagram of the synthesis of Mg(OH)_2_ nanoflakes.

A direct precipitation method was used to synthesize Mg(OH)_2_ nanoflakes in an aqueous solution at room temperature (Figure 1). This synthesis process involved precipitating Mg(OH)_2_ in an alkaline environment without a capping agent, yielding pure, effective materials. The technique serves as a very economical, safe, and straightforward approach to preparing Mg(OH)_2_ nanoflakes. This synthesis approach is simple, faster, uses low energy, and has a low aging duration compared to other investigations, which are energy‐intensive, such as hydrothermal [23], heating at 90°C [24], heating at 85°C [25], and a high aging period of 24 h [26].

**FIGURE 2:**
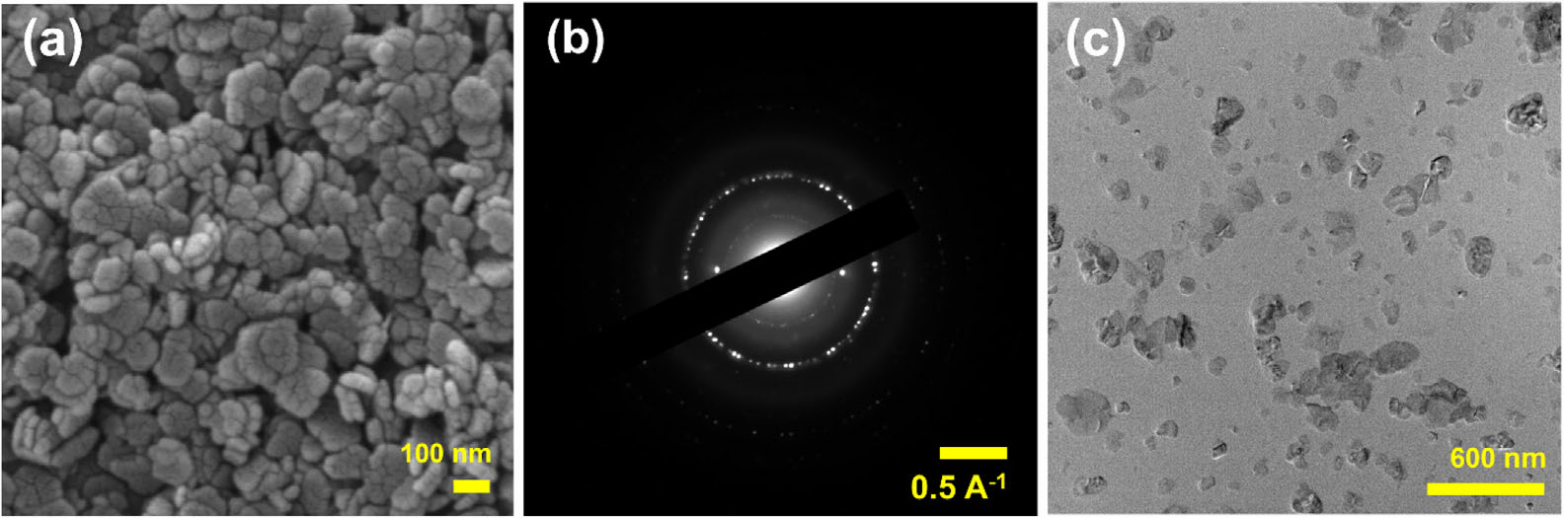
(a) FESEM, (b) SAED, and (c) TEM images of Mg(OH)_2_ nanoflakes.

FESEM images (Figure 2a) revealed segregated nanoflakes that appear loosely joined together. ImageJ size analysis revealed particle diameters ranging from about 50 nm to 140 nm as shown in Figure S1, with each particle showing multiple fragmentations, exposing more active sites for adequate adsorption. TEM (Figure 2c) further confirms the formation of thin nanoflakes of Mg(OH)_2_. The scanning transmission electron microscopy, together with the EDX analysis (Figures S2), confirms the atomic percentages of 46.2% and 53.8% for Mg and O, respectively, in the sample, which are very close to theoretical values. The distinct ring with many brighter spots in the selected‐area electron diffraction (SAED) pattern (Figure 2b) corresponds to Bragg's reflection, confirming the polycrystalline nature of Mg(OH)_2_.

**FIGURE 3:**
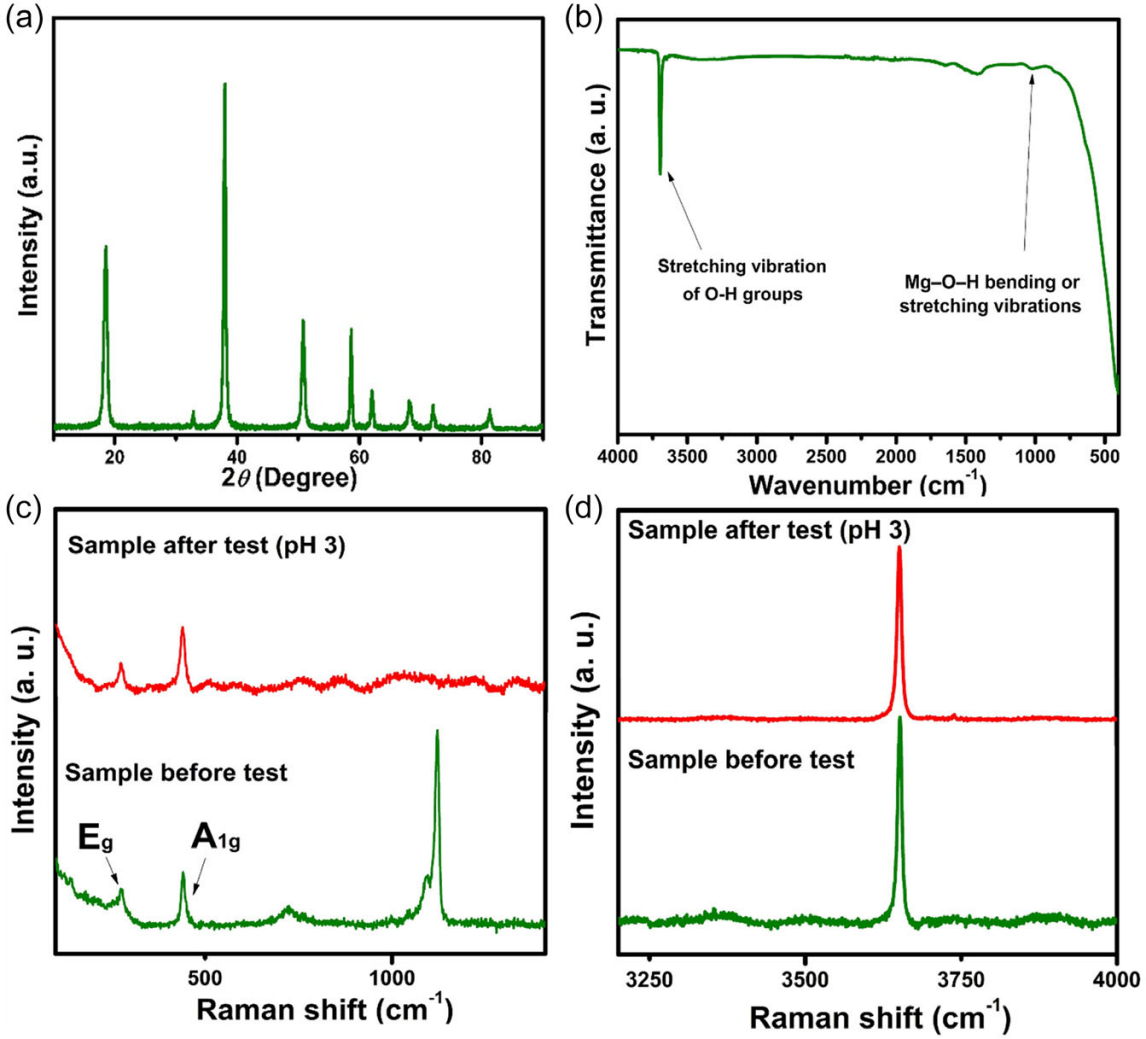
Mg(OH)_2_ spectra obtained from (a) XRD analysis and (b) FTIR analysis before the adsorption test. (c, d) Raman spectra before and after adsorption test.

The crystallinity of the nanoflakes was studied using XRD (Figure 3a) which revealed diffraction patterns matched with the brucite hexagonal Mg(OH)_2_ (PDF no. 01‐071−5972). Peaks observed at 2*θ* angles of 18.57^o^, 32.80^o^, 37.95^o^, 50.80^o^, 58.52^o^, 62.00^o^, 68.19^o^, 71.92^o^, and 81.15^o^ corresponded to the (001), (100), (011), (012), (110), (111), (103), (201), and (202) lattice planes, respectively. FTIR studies (Figure 3b) showed a sharp, intense peak at about 3694 cm^−1^, signifying the stretching vibrations of the O—H group from the Mg(OH)_2_ nanoflakes, which are not bonded to water molecules. These non‐hydrogen‐bonded O—H groups are hydroxyl (O—H) groups in a molecule that are not involved in hydrogen bonding interactions with other molecules [27]. Hydrogen‐bonded O—H groups show a broad O—H stretching vibration at about 3200–3400 cm^−1^. A peak at 1007 cm^−1^ signifies Mg—O—H bending or stretching vibrations. From the spectrum, a peak observed at 1416 cm^−1^ can be attributed to carbonate (CO_3_^2−^) ions [28] from adsorbed CO_2_ in the sample during FTIR measurements. A broad peak is observed at 3200–3400 cm^−1^ for the O—H stretching vibration, and a very narrow peak at 1645 cm^−1^, indicative of H—O—H bending, indicating low moisture adsorption.

Structurally, one Mg^2+^ ion linking two OH^‐^ groups per formula unit gives magnesium hydroxide a five‐atom unit cell [29]. This unit cell is derived from the hexagonal crystal layered system. The formation of sheet‐like morphology can be attributed to a two‐dimensional nucleation and growth technique, where the Mg^2+^ ions are coordinated by OH^−^ ions octahedrally, resulting in brucite‐like layers stacked via hydrogen bonding [30, 31]. With a D_3d_ point group as derived from Mg(OH)_2_ molecular symmetry, A_1g_ and E_g_ Raman‐active vibrational modes are expected in the Raman spectrum of Mg(OH)_2_ [29, 32]. However, other bands are primarily observed in the high‐wave‐number region due to hydroxyl stretching. The two expected Raman bands were observed at low‐wave‐number vibrations of about 277.7 cm^−1^ and 441.2 cm^−1^ for the sample before the adsorption test (Figure 3c). A high‐wave‐number mode which is known to occur in the region [32] of around 3600 cm^−1^ to 3800 cm^−1^ was also observed at 3651.6 cm^−1^ (Figure 3d). This vibrational mode is mainly attributed to a strong symmetric O—H stretch. The vibrational band produced at 1121.7 can be attributed to the symmetrical stretching [32] of the carbonate ion (CO_3_^2‐^). The absence of bands from carbonyl groups after the adsorption test (Figure 3c) can be attributed to weak bonding and to their diffusion into solution, as they were absent in the samples.

**FIGURE 4:**
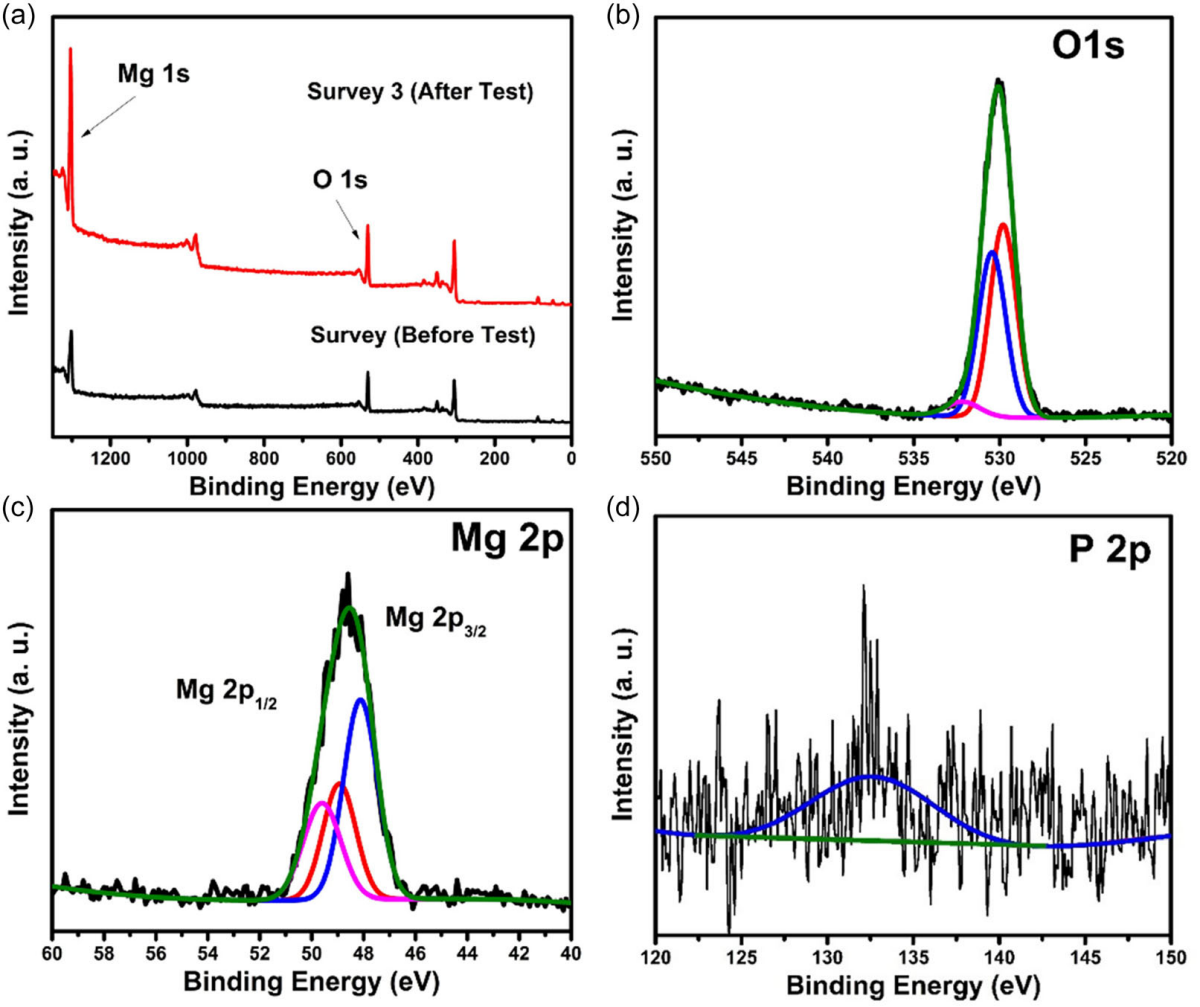
(a) XPS survey spectrum showing the presence of Mg and O, (b) O 1s spectrum, (c) Mg 2p spectrum, and (d) P 2p spectrum after the adsorption test.

XPS analysis before the adsorption test revealed the presence of Mg and O in the survey (Figure 4a). Results after PO_4_^3−^ adsorption test showed the presence of phosphorus (P 2p at 132.6 eV) from the recovered nanoflakes, which confirms a successful adsorption process. There is a shift in the Mg 1s peak (Figure S3c) after the adsorption test, indicating some degree of chemical interaction between the P and the Mg. Deconvoluted O 1s peaks also resulted in a slight shift of binding energy before and after the adsorption test (Figures 4b and S3b). The change in Mg binding energy after the adsorption test is due to the chemical environment created by phosphate interaction, indicating that Mg serves as an active adsorption site. The deconvoluted Mg 2p peak before adsorption showed the presence of a peak at 50.46 eV [33], indicating the presence of Mg(OH)_2_. This binding energy shifted to a lower binding energy level after the adsorption test (Figure 4c), with a reduced peak area indicating the consumption of Mg(OH)_2_ and the formation of a new chemical environment due to the formation of Mg_3_(PO_4_)_2_. The P 2p was not deconvoluted due to the low intensity, to investigate the presence of HPO_4_^2−^ expected at 133.8 eV [34].

**FIGURE 5:**
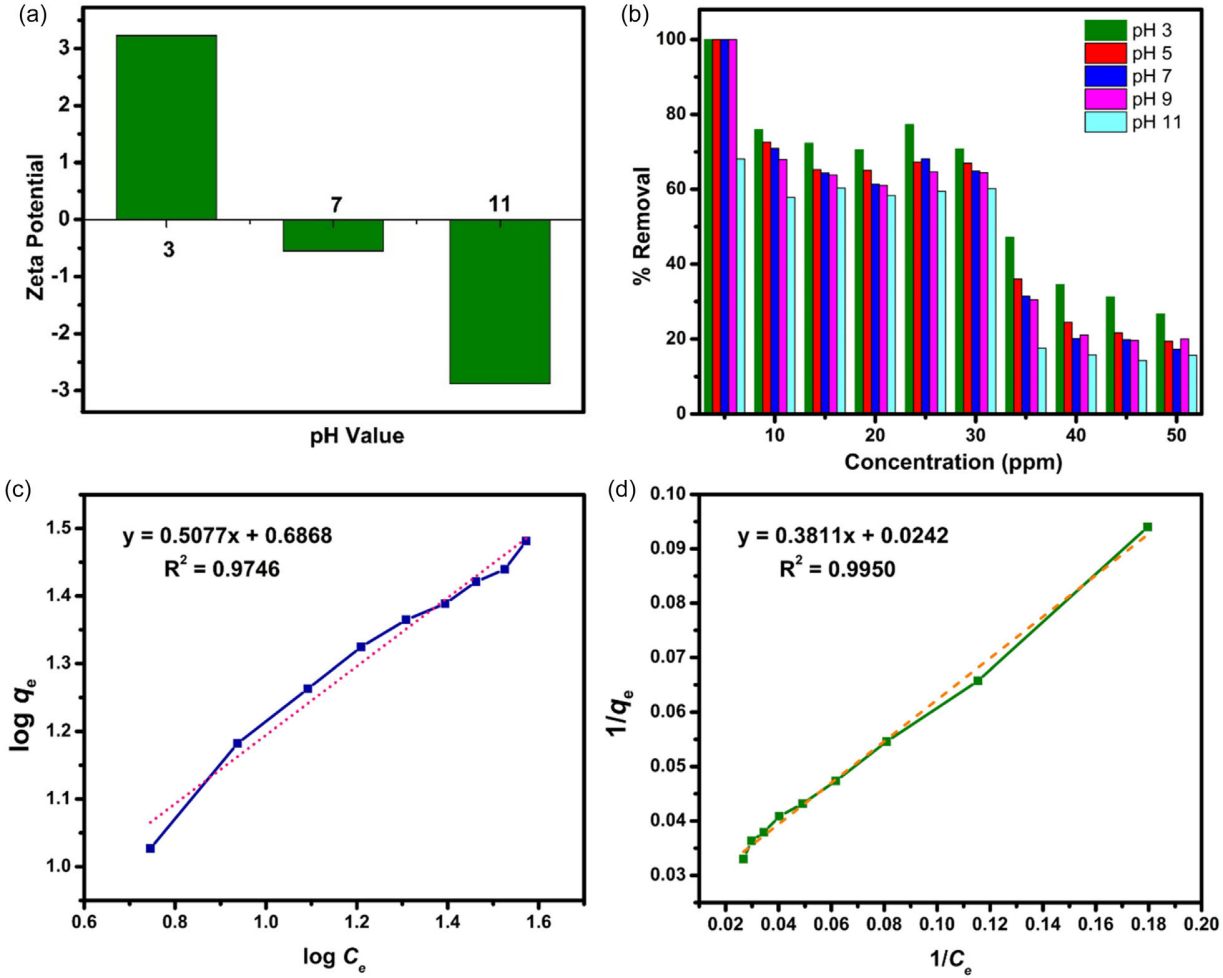
(a) Zeta potential analysis carried out at pH 3, 7, and 11; (b) experimental adsorption test results at different concentrations and different pH values. Determination of (c) Freundlich and (d) Langmuir isotherms of the phosphate ions adsorption.

To investigate the surface charges of the prepared NPs and their effects on adsorption, zeta potential analysis was performed. Zeta potential analysis was performed at pH 3, 7, and 11 for the sample before the adsorption test, revealing a highly positive surface charge at pH 3 (Figure 5a) compared to pH 7 and 11. The negative surface charge increased with increasing pH. pH 3 resulted in a zeta potential of 3.2 mV, −0.5 mV at pH 7, and −2.8 mV at pH 11. Cationic behavior at pH 3 significantly enhanced the attraction of anionic phosphate ions from solution, thereby yielding higher adsorption (Figure 5b). The negative surface charge at pH 11, as obtained from zeta potential, may have induced some degree of repulsion from the phosphate ions.

Results from the phosphate ion adsorption test showed higher phosphate removal at pH 3 across all phosphate solutions than at any other pH. This can be attributed to the effectiveness of the nanoparticles and their positively charged surfaces, as observed in the zeta potential analysis. The mechanism of the adsorption process is characterized by initial physisorption through ionic attraction, followed by chemisorption, where some adsorbed ions chemically interact with Mg to form Mg_3_(PO_4_)_2_.

The Langmuir and Freundlich adsorption isotherms, which model the interaction between the adsorbate and the adsorbent during adsorption, were tested to characterize the adsorption behavior of phosphate ions on Mg(OH)_2_. The two isotherm equations for the adsorption process were fitted to the experimental results. The Langmuir and the Freundlich nonlinear equations were represented by Equations (3) and (4).

The isotherm was fitted using the equation:

(3) $$q_{e}=\frac{q_{m}K_{L}C_{e}}{1+K_{L}C_{e}}$$

(4) $$q_{e}=K_{f}{C_{e}}^{1/n}$$

where *q*_*m*_ is the maximum adsorption capacity in mg/g, *K*_*L*_ is the Langmuir constant in L/mg, and K_*f*_ in mg/g and *n* are the Freundlich constants. The equilibrium concentration of adsorbate is represented by *C*_e_, while the amount adsorbed per unit mass is given by *q*_*e*_.

The experimental results showed that both models fit the phosphate adsorption data excellently. For the Langmuir isotherm, the linear plot of 1/*Q*_e_ versus 1/*C*_e_ yielded the equation *y* = 0.3811x + 0.0242 with an outstanding correlation coefficient of *R* ^2^ = 0.9950. From the y‐intercept (1/*Q*_*max*_ = 0.0242), the maximum adsorption capacity was calculated as *Q*_*max*_ = 41.32 mg/g, indicating that the Mg(OH)_2_ surface can accommodate this amount of phosphate in a complete monolayer. From the slope [1/(*Q*_*max*_ × *K*_*L*_) = 0.3811], the Langmuir constant was determined to be *K*_*L*_ = 0.0635 L/mg, which reflects moderate binding affinity between phosphate and the adsorbent surface. The dimensionless separation factor (*R*_*L*_) was calculated using *R*_*L*_ = 1/(1 + *K*_*L*_ × *C*_*0*_) for various initial concentrations, yielding values ranging from 0.136 to 0.612, all of which fall between 0 and 1, confirming that phosphate adsorption on Mg(OH)_2_ is favorable across the entire concentration range studied. The Freundlich isotherm analysis, plotting log Q_e_ versus log C_e_, produced the linear equation *y* = 0.5077x + 0.6868 with R^2^ = 0.9746. The slope (1/*n* = 0.5077) resulted in *n* = 1.97. Since *n* > 1 (specifically between 1 and 10), this indicates favorable adsorption. The y‐intercept (log *K*_*f*_ = 0.6868) yielded *K*_*f*_ = 4.86 (mg/g)(L/mg)^1/*n*^, representing the adsorption capacity and bond strength of the system.

As shown in Figures 5c and 5d, the *R* ^2^ values obtained from the linear plots of the Freundlich model and the Langmuir model of the adsorption of the phosphate ions were 0.9746 and 0.9950, respectively. Comparing the two models, the Langmuir isotherm provided a marginally better fit (*R* ^2^ = 0.9950) compared to the Freundlich model (*R* ^2^ = 0.9746), though both correlations are excellent. The adsorption process can therefore be said to closely follow the Langmuir isotherm model. The superior Langmuir fit suggests that phosphate adsorption on Mg(OH)_2_ occurs predominantly through monolayer coverage on relatively homogeneous binding sites, consistent with the formation of surface complexes between phosphate ions and magnesium sites. The exhibition of monolayer homogeneous adsorption by the Langmuir model suggests no interaction or reaction between adsorbates [35]. As a result, it can be inferred that there is only monolayer adsorption and that all adsorption sites are equivalent and have uniform energy. The results also revealed that the phosphate adsorption on the Mg(OH)_2_ NPs occurs consistently until all surfaces reach complete saturation.

The strong performance of the Freundlich model indicates that some degree of surface heterogeneity exists, which is reasonable given that Mg(OH)_2_ nanoparticles present different types of sites, such as edge sites, basal plane sites, and surface defects, each potentially having different binding energies. The mechanism for the highest adsorption at pH 3 can be attributed to possible physical adsorption where species from the liquid phase accumulate on the surface of the solid prior to the onset electrostatic interaction. At pH 3, the phosphate will exist in solution predominantly in its protonated forms, mainly as H_3_PO_4_ or H_2_PO_4_^−^. Mg(OH)_2_ with its low solubility constant (Ksp ≈ 4.8 × 10^−12^) [36] will largely dissolve (Equation (5)) due to water and acid neutralization releasing Mg^2+^ ions into solution where aqueous inner sphere complexation with phosphate species like H_2_PO_4_^−^ can occur forming monodentate complex (Equation (6)) via direct Mg–O–P bonding [37].

(5) $$\text{Mg}{\left(\text{OH}\right)}_{2(s)}+2H^{+}\to \;{\text{Mg}}^{2+}+2H_{2}O$$

(6) $${\text{Mg}}^{2+}+H_{2}{\text{PO}}_{4}^{-}⇌\text{Mg}H_{2}{\text{PO}}_{4}^{+}$$

Recycle test performed on the Mg(OH)_2_ sample recovered at pH 3 showed low performance (Figure S5). This confirms that Mg(OH)_2_ is poorly recovered at pH 3 after phosphate adsorption as the acidic condition promotes its dissolution into soluble Mg^2+^ ions rather than precipitation. Only 11.1 mg of solids was recovered after the first phosphate adsorption analysis and 9.3 mg recovered after the second analysis. These weights were used in the second and third cycles, respectively, (Figure S5). The reduced weights used in the recovery also contributed to the low recovery yield. Even though the acidic condition has proven more effective, good recovery is generally achieved via adsorption in the pH 9–11 conditions. This occurs through the surface adsorption and precipitation mechanisms as outlined in Equation (7).

(7) $$\equiv \text{Mg}-\text{OH}+{\text{HPO}}_{4}^{2-}\to \equiv \text{Mg}-{\text{PO}}_{4}+{\text{OH}}^{-}$$

At moderately basic pH (8–10), the Mg(OH)_2_ is dispersed in solution. The presence of Mg^2+^ ions on the surface of the Mg(OH)_2_ facilitates the adsorption of phosphate ions (PO_4_^3−^, HPO_4_^2−^, or H_2_PO_4_^−^) from the solution via electrostatic attraction. Ligand exchange then forms inner‐sphere complexes, where phosphate binds directly to Mg sites, replacing surface OH groups. Excess OH^−^ competes with phosphate increasing surface negative charges, thereby decreasing adsorption efficiency followed by precipitation.^[^ ^38^ ^,^ ^39^ ^]^ Based on the adsorption mechanism, the stability of magnesium hydroxide (Mg(OH)_2_) during phosphate adsorption can be attributed to its strong dependence on pH and ionic strength. Mg(OH)_2_ is more stable at high pH; however, it shows low adsorption. The long‐term stability of the Mg(OH)_2_ nanoflakes can be achieved by alkaline desorption technique where the phosphate loaded material is treatment with NaOH, centrifuged, washed, and dried between 60°C and 105°C [39]. The Langmuir constant (*K*_*L*_ = 0.0635 L/mg) indicates moderate binding affinity, which is advantageous for practical applications as it suggests stable adsorption without being so strong as to prevent adsorbent regeneration. The Freundlich parameter *n* = 1.97 further supports favorable adsorption conditions, as values between 1 and 10 typically indicate beneficial adsorption characteristics.

From a practical standpoint, the favorable *R*_*L*_ values indicate that the adsorbent will perform effectively across a wide range of phosphate concentrations commonly encountered in wastewater streams. Using the Langmuir equation, the adsorbent dose required for effective treatment can be estimated. For example, achieving 90% removal of phosphate from wastewater with an initial concentration of 20 mg/L would require ≈ 3.6 g of adsorbent per liter of wastewater. The success of both isothermal models provides confidence in the reliability of the adsorption data and offers complementary insights into the adsorption process. The Langmuir model's superior fit confirms that monolayer adsorption is the primary mechanism, while the Freundlich model captures the energetic heterogeneity inherent in real nanoparticle surfaces. Overall, both isotherm analyses converge to demonstrate that Mg(OH)_2_ is an effective and promising adsorbent for phosphate removal from aqueous solutions, with high capacity, favorable thermodynamics, and a well‐defined adsorption mechanism suitable for wastewater treatment applications.

Using goethite as adsorbent at pH 8, Chitrakar et al. reported Cl^−^ < NO^3−^ < SO_4_^2−^ << CO_3_^2−^ < HPO_4_^2−^ as the order of selectivity of phosphate adsorption [40]. Geelhoed et al. confirmed the higher selectivity of phosphate ions to sulfate, even though sulfate can form inner‐sphere complexes [41, 42]. Other investigations have reported preferential adsorption of phosphate in the presence of carbonate ions. Even though carbonates have comparatively low selectivity, they lower the electrophoretic mobility and may reduce the adsorbent capacity, attributed to inner‐sphere carbonate adsorption [42]. Using Mg(OH)_2_ particles, anions such as anions Cl^−^, and NO_3_^−^ have been reported to interact weakly, forming outer sphere complexes and usually exhibits minor effect on phosphate adsorption on metal hydroxides or layered Mg materials [43]. SO_4_^2−^ and HCO_3_^−^ ions can possibly form stronger surface complexes and at higher concentrations can significantly compete with phosphate for adsorption sites [43]. This confirms that Mg(OH)_2_ is selective toward phosphate ions, and further minimization of interference from competing ions in real‐time wastewater analysis can be achieved via pH adjustment and other techniques, such as surface modification to improve phosphate affinity and pre‐removal or suppression techniques toward possible interfering species.

To investigate the state of the absorbent after the adsorption process, FTIR and XRD analyses were performed. As shown in Figure S6, the O—H stretching vibration due to non‐hydrogen‐bonded O—H groups is hydroxyl (O—H) groups at a wave number of about 3694 cm^−1^ for the sample after adsorption, which is less intense compared to the sample before adsorption. This confirms that the O—H groups are being consumed during the adsorption. Peaks at about 1024 and 1651 cm^−1^ were intensified in the samples after the adsorption test. The 1024 cm^−1^ peak can be attributed to the asymmetric stretching vibration of PO_4_^3−^ [44, 45]. XRD pattern showed an increase in intensity in the peak at 18.57° for all samples (Figure S7) after the adsorption test. A decreased intensity in peak at 37.95° occurred in all samples after the adsorption test compared to the Mg(OH)_2_. This shows that the adsorption influenced the crystallinity of the pure Mg(OH)_2_. However, the absence of peaks from the expected formation of Mg_3_(PO_4_)_2_ can be attributed to its low concentration. The absence of the peak at 32.80° can also be attributed to the influence of the adsorbed phosphate species. Post‐adsorption characterization was performed after the samples had been recovered via centrifugation, decantation of supernatant solution, and well dried in the oven at 60°C until dry showing the stability of the adsorbent.

## 4 Conclusions

In this study, Mg(OH)_2_ nanoflakes were successfully synthesized using the precipitation method without the use of surfactants and capping agents. Zeta potential measurements indicated a positive surface charge of nanoflakes for the materials dispersed in a solution adjusted to a pH of 3. Mg(OH)_2_ in a solution at pH 3 showed the highest phosphate removal efficiency from the solution, which can be attributed to additional electrostatic interactions from the positively charged surfaces and the negatively charged phosphate ions. As observed in zeta potential, the sample with pH 11 exhibited a higher negative surface charge and relatively low phosphate removal, which can be attributed to some degree of repulsion from the phosphate ions. The Langmuir isotherm, which assumes monolayer adsorption on a homogeneous surface with identical binding sites, where each site can hold only one adsorbate molecule with no interaction between adsorbed species, was identified as the best fit for the adsorption process. An outstanding *R* ^2^ of 0.9950 was obtained for the model. The straightforward, single‐step, low‐temperature synthesis method provides a cost‐effective option for the fabrication of the materials used in this wastewater treatment study. The higher phosphate removal rate makes Mg(OH)_2_ nanoflakes effective for phosphate ion removal in water treatment applications.

## Supporting Information

Additional supporting information can be found online in the Supporting Information section. EDS images, XPS spectra before the adsorption test, zeta potential plot, FTIR, and XRD before and after the adsorption test. The supporting information is attached.

## Author Contributions

**M.A.**: formal analysis and investigation, methodology, writing – original draft preparation, writing – review and editing. **N.C.**: formal analysis and investigation, methodology, writing – original draft preparation, writing – review and editing. **S.R.**: formal analysis and investigation, methodology, writing – original draft preparation, writing – review and editing. **N.A.: s**upervision, writing – original draft preparation, writing – review and editing, funding acquisition. **B.P.B.**: **c**onceptualization, supervision, writing – original draft preparation, writing – review and editing.

## Funding

This work was supported by the USDA NIFA Evans Allen, project award no. NC.X333‐5‐21‐130‐1, from the U.S. Department of Agriculture's National Institute of Food and Agriculture.

## Conflicts of Interest

The authors declare no conflicts of interest.

## Supporting information

Supplementary Material

## Data Availability

All data are used in the manuscript and supporting information

## References

[1] United Nations Environment Programme , What Is Phosphorus and Why Are Concerns Mounting About Its Environmental Impact?,” UNEP 2024.

[2] SNEP Network , “All About Phosphorus., 2024, https://snepnetwork.org/all‐about‐phosphorus/.

[3] T. E. Higham , Phosphorus Cycle, in Encyclopedia of Toxicology, 2014, pp. 952–961, 10.1016/B978-0-1237-4553-8.00216-1.

[4] S. Singh , S. Jain , V. Ps , et al., “Hydrogen: A Sustainable Fuel for Future of the Transport Sector,” Renewable and Sustainable Energy Reviews 51 (2015): 623–633, 10.1016/j.rser.2015.06.040.

[5] N. Schwartz , “Water Quality, Streamflow, and Ancillary Data for Nutrients in Streams and Rivers Across the Nation,” Journal of Religion in Africa 35 (2005): 159–196, https://brill.com/view/journals/jra/35/2/article‐p159_4.xml

[6] K. Vikrant , K. H. Kim , Y. S. Ok , et al., “Engineered/Designer Biochar for the Removal of Phosphate in Water and Wastewater,” Science of the Total Environment 616‐617 (2018): 1242–1260, 10.1016/j.scitotenv.2017.10.193. 29107379

[7] US Geological Survey (USGS) , Eutrophication.,” U.S. Geological Survey. (1999).

[8] N. A. Serediak , E. E. Prepas , and G. J. Putz , Eutrophication of Freshwater Systems, in Treatise on Geochemistry, 2014, pp. 305, 2nd, 11, 10.1016/B978-0-08-095975-7.00908-6.

[9] H. Xu , S. Wei , G. Li , and B. Guo , “Advanced Removal of Phosphorus from Urban Sewage Using Chemical Precipitation by Fe–Al Composite Coagulants,” Scientific Reports 14 (2024): 1–12, 10.1038/s41598-024-55713-2. 38418598 PMC10901887

[10] K. B. Zare and M. Tanwer , “Exploring Effective Strategies for Phosphorus Removal from Wastewater: A Comprehensive Review of Chemical, Biological, and Physicochemical Methods,” Research Journal of Recent Sciences 14 (2025): 25–34, https://www.isca.me/rjrs/archive/v14/i1/4.ISCA‐RJRS‐2024‐021.php

[11] S. S. N. Team , “Phosphorus Removal from Wastewater.”, Seven Seas Water Corp, 2024, https://sevenseaswater.com/phosphorus‐removal‐from‐wastewater/.

[12] P. J. A. Borm , D. Robbins , S. Haubold , et al., “The Potential Risks of Nanomaterials: A Review Carried Out for ECETOC,” Particle and Fibre Toxicology 3 (2006): 11, 10.1186/1743-8977-3-11. 16907977 PMC1584248

[13] Y. Wang , J. Lin , Y. Wang , Z. Liu , J. Lian , and M. Liu , “Highly Efficient and Selective Removal of Low‐Concentration Antibiotics from Aqueous Solution by Regenerable Mg(OH)_2_ ,” Journal of Environmental Sciences 87 (2020): 228–237, 10.1016/j.jes.2019.06.017. 31791495

[14] S. Munkaila , R. Dahal , M. Kokayi , T. Jackson , and B. P. Bastakoti , “Hollow Structured Transition Metal Phosphates and Their Applications,” The Chemical Record 22 (2022): e202200084, 10.1002/tcr.202200084. 35815949

[15] O. Falyouna , K. Bensaida , I. Maamoun , et al., “Synthesis of Hybrid Magnesium Hydroxide/Magnesium Oxide Nanorods [Mg(OH)_2_/MgO] for Prompt and Efficient Adsorption of Ciprofloxacin from Aqueous Solutions,” Journal of Cleaner Production 342 (2022): 130949, 10.1016/j.jclepro.2022.130949.

[16] X. Guo , J. Lu , and L. Zhang , “Magnesium Hydroxide with Higher Adsorption Capacity for Effective Removal of Co(II) from Aqueous Solutions,” Journal of the Taiwan Institute of Chemical Engineers 44 (2013): 630–636, 10.1016/j.jtice.2012.12.020.

[17] R. Orij , S. Brul , and G. J. Smits , “Intracellular pH Is a Tightly Controlled Signal in Yeast,” Biochimica et Biophysica Acta – General Subjects 1810 (2011): 933–944, 10.1016/j.bbagen.2011.03.011. 21421024

[18] Y. Yang , D. Liu , Y. Chen , J. He , and Q. Li , “Mechanistic Study of Highly Effective Phosphate Removal from Aqueous Solutions over a New Lanthanum Carbonate Fabricated Carbon Nanotube Film,” Journal of Environmental Management 359 (2024): 120938, 10.1016/j.jenvman.2024.120938. 38669888

[19] S. Munkaila , J. Bentley , K. Schimmel , T. Ahamad , S. M. Alshehri , and B. P. Bastakoti , “Polymer Directed Synthesis of NiO Nanoflowers to Remove Pollutant from Wastewater,” Journal of Molecular Liquids 324 (2021): 114676, 10.1016/j.molliq.2020.114676.

[20] M. K. Bhattarai , M. D. Ashie , S. Dugu , et al., “Block Copolymer‐Assisted Synthesis of Iron Oxide Nanoparticles for Effective Removal of Congo Red,” Molecules (basel, Switzerland) 28 (2023), 10.3390/molecules28041914. PMC9964741 36838902

[21] B. P. Bastakoti , N. Bhattarai , M. D. Ashie , F. Tettey , S. I. Yusa , and K. Nakashima , “Single‐Micelle‐Templated Synthesis of Hollow Barium Carbonate Nanoparticle for Drug Delivery,” Polymers 15 (2023): 1739, 10.3390/polym15071739. 37050353 PMC10096637

[22] B. P. Bastakoti , S. Munkaila , and S. Guragain , “Micelles Template for the Synthesis of Hollow Nickel Phosphate Nanospheres,” Materials Letters 251 (2019): 34–36, 10.1016/j.matlet.2019.05.034.

[23] J. M. Hanlon , L. B. Diaz , G. Balducci , et al., “Rapid Surfactant‐Free Synthesis of Mg(OH)_2_ Nanoplates and Pseudomorphic Dehydration to MgO,” CrystEngComm 17 (2015): 5672, 10.1039/c5ce00595g.

[24] R. Giorgi , C. Bozzi , L. Dei , C. Gabbiani , B. W. Ninham , and P. Baglioni , “Nanoparticles of Mg(OH)_2_: Synthesis and Application to Paper Conservation,” Langmuir : the Acs Journal of Surfaces and Colloids 21 (2005): 8495–8501, 10.1021/la050564m. 16114962

[25] H. Dong , Z. Du , Y. Zhao , and D. Zhou , “Preparation of Surface Modified Nano‐Mg(OH)_2_ via Precipitation Method,” Powder Technology 198 (2010): 325–329, 10.1016/j.powtec.2009.11.014.

[26] J. Hsu and A. Nacu , “Preparation of Submicron‐Sized Mg(OH)_2_ Particles through Precipitation,” Colloids and Surfaces A: Physicochemical and Engineering Aspects 262 (2005): 220–231, 10.1016/j.colsurfa.2005.04.038.

[27] F. Tang , T. Ohto , T. Hasegawa , et al., “Definition of Free O–H Groups of Water at the Air–Water Interface,” Journal of Chemical Theory and Computation 14 (2018): 357–364, 10.1021/acs.jctc.7b00566. 29156124

[28] S. Veerasingam and R. Venkatachalapathy , “Estimation of Carbonate Concentration and Characterization of Marine Sediments by Fourier Transform Infrared Spectroscopy,” Infrared Physics and Technology 66 (2014): 136–140, 10.1016/j.infrared.2014.06.005.

[29] A. Suslu , K. Wu , H. Sahin , et al., “Unusual Dimensionality Effects and Surface Charge Density in 2D Mg(OH)_2_ ,” Scientific Reports 6 (2016): 20525, 10.1038/srep20525. 26846617 PMC4742812

[30] H. Cao , H. Zheng , J. Yin , et al., “Mg(OH)_2_ Complex Nanostructures with Superhydrophobicity and Flame Retardant Effects,” Journal of Physical Chemistry C 114 (2010): 17362–17368, 10.1021/jp107216z.

[31] G. Balducci , L. Bravo Diaz , and D. H. Gregory , “Recent Progress in the Synthesis of Nanostructured Magnesium Hydroxide,” CrystEngComm 19 (2017): 6067–6084, 10.1039/c7ce01570d.

[32] M. Dekermenjian , A. P. Ruediger , and A. Merlen , “Raman Spectroscopy Investigation of Magnesium Oxide Nanoparticles,” RSC Advances 13 (2023): 26683–26689, 10.1039/d3ra04492k. 37681036 PMC10481257

[33] S. A. Skaanvik , J. D. Henderson , J. J. Noël , and M. C. Biesinger , “Speciation of Magnesium Surfaces by X‐Ray Photoelectron Spectroscopy (XPS),” Surface and Interface Analysis 57 (2025): 717–728, 10.1002/sia.70003.

[34] F. Xie , F. Wu , G. Liu , et al., “Removal of Phosphate from Eutrophic Lakes through Adsorption by In Situ Formation of Magnesium Hydroxide from Diatomite,” Environmental Science & Technology 48 (2014): 582–590, 10.1021/es4037379. 24328241

[35] H. Swenson and N. P. Stadie , “ *Langmuir*'s Theory of Adsorption: A Centennial Review,” Langmuir : the Acs Journal of Surfaces and Colloids 35 (2019): 5409–5426, 10.1021/acs.langmuir.9b00154. 30912949

[36] A. A. Al‐Hamzah , E. J. Smith , and C. M. Fellows , “Inhibition of Homogeneous Formation of Magnesium Hydroxide by Low‐Molar‐Mass Poly(acrylic Acid) with Different End‐Groups,” Industrial & Engineering Chemistry Research 54 (2015): 2201–2207, 10.1021/ie504869e.

[37] B. Koca , M. Jafari , L. F. Song , Z. Li , V. Aviyente , and K. M. Merz , “Binding of Phosphate Species to Ca^2+^ and Mg^2+^ in Aqueous Solution,” Journal of Chemical Theory and Computation 20 (2024): 4298–4307, 10.1021/acs.jctc.4c00218. 38718258 PMC11137831

[38] F. Bu , L. Han , H. Guo , Y. Liang , and H. Yan , “Unraveling the Synergistic Mechanisms of Phosphorus Adsorption and Slow‐Release on Low‐Mg‐Loaded Biochar Enabled by KOH Activation,” Materials 18 (2025): 5214, 10.3390/ma18225214. 41304058 PMC12654471

[39] P. Tu , G. Zhang , Y. Cen , et al., “Enhanced Phosphate Adsorption and Desorption Characteristics of MgO‐Modified Biochars Prepared via Direct Co‐Pyrolysis of MgO and Raw Materials,” Bioresources and Bioprocessing 10 (2023): 49, 10.1186/s40643-023-00670-3. 38647775 PMC10991339

[40] R. Chitrakar , S. Tezuka , A. Sonoda , K. Sakane , K. Ooi , and T. Hirotsu , “Phosphate Adsorption on Synthetic Goethite and Akaganeite,” Journal of Colloid and Interface Science 298 (2006): 602–608, 10.1016/j.jcis.2005.12.054. 16455102

[41] J. S. Geelhoed , T. Hiemstra , and W. H. Van Riemsdijk , “Phosphate and Sulfate Adsorption on Goethite: Single Anion and Competitive Adsorption,” Geochimica et Cosmochimica Acta 61 (1997): 2389–2396, 10.1016/S0016-7037(97)00096-3.

[42] G. Ferric , H. Gfh , T. Reinhardt , et al., “Batch Studies of Phosphonate and Phosphate Membrane Concentrate and Its Synthetic Replicas,” Molecules (basel, Switzerland) 25 (2020): 5202, 10.3390/molecules25215202. 33182263 PMC7664883

[43] M. N. Nadagouda , G. Varshney , V. Varshney , and C. A. Hejase , “Recent Advances in Technologies for Phosphate Removal and Recovery: A Review,” ACS Environmental Au 4 (2024): 271–291, 10.1021/acsenvironau.3c00069. 39582759 PMC11583102

[44] V. Andrushchenko , L. Benda , O. Páv , M. Dračínský , and P. Bouř , “Vibrational Properties of the Phosphate Group Investigated by Molecular Dynamics and Density Functional Theory,” Journal of Physical Chemistry B 119 (2015): 10682–10692, 10.1021/acs.jpcb.5b05124. 26193890

[45] P. Pettersson and A. Barth , “Correlations between the Structure and the Vibrational Spectrum of the Phosphate Group: Implications for the Analysis of an Important Functional Group in Phosphoproteins,” RSC Advances 10 (2020): 4715–4724, 10.1039/c9ra10366j. 35495230 PMC9049017

