# Supplementary material for: Mg(OH)2 Nanoflakes for Effective Removal of Phosphate Ions
Source: ChemistryOpen. 2026 Apr 17;15(5):e70188. doi: 10.1002/open.70188 (PMC13087892; doi:10.1002/open.70188)
Supplement: Supplementary file 1 — Supplementary Material [file OPEN-15-e70188-s001.pdf]

$Mg(OH)_2$ Nanoflakes for Effective Removal of Phosphate Ions

Moses Ashie[1], Nicholas Chance[2], Sushmita Roy[2], Niroj Aryal[2]*, Bishnu Prasad Bastakoti[1]*

[1]Department of Chemistry, North Carolina A&T State University, Greensboro, NC, 27411, USA

[2]Department of Natural Resources and Environmental Design, North Carolina A&T State University, Greensboro, NC, 27411, USA

naryal@ncat.edu and bpbastakoti@ncat.edu

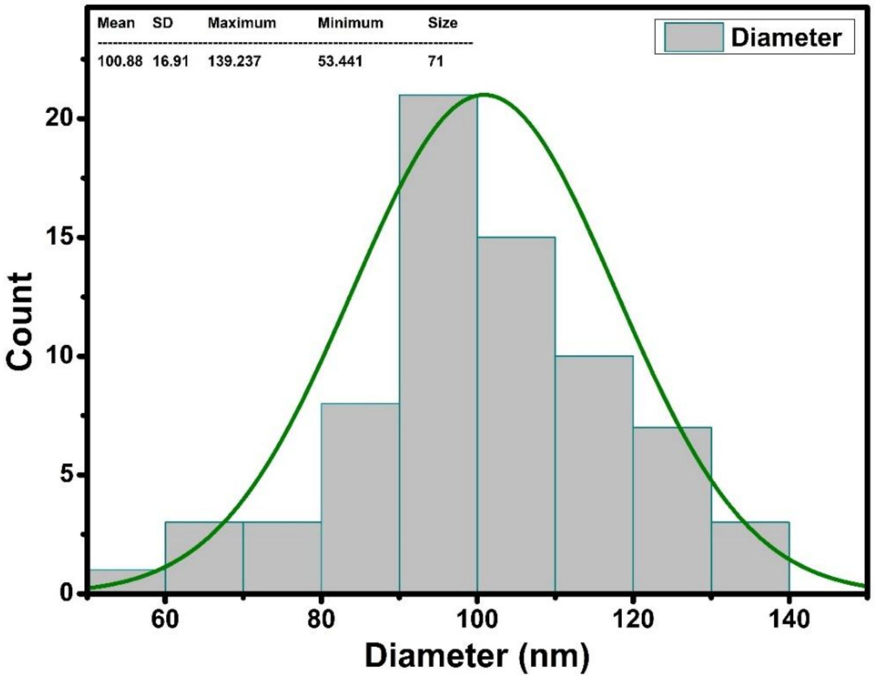

Figure S1: A histogram showing the particle size distribution of the fabricated $Mg(OH)_2$ nanoflakes

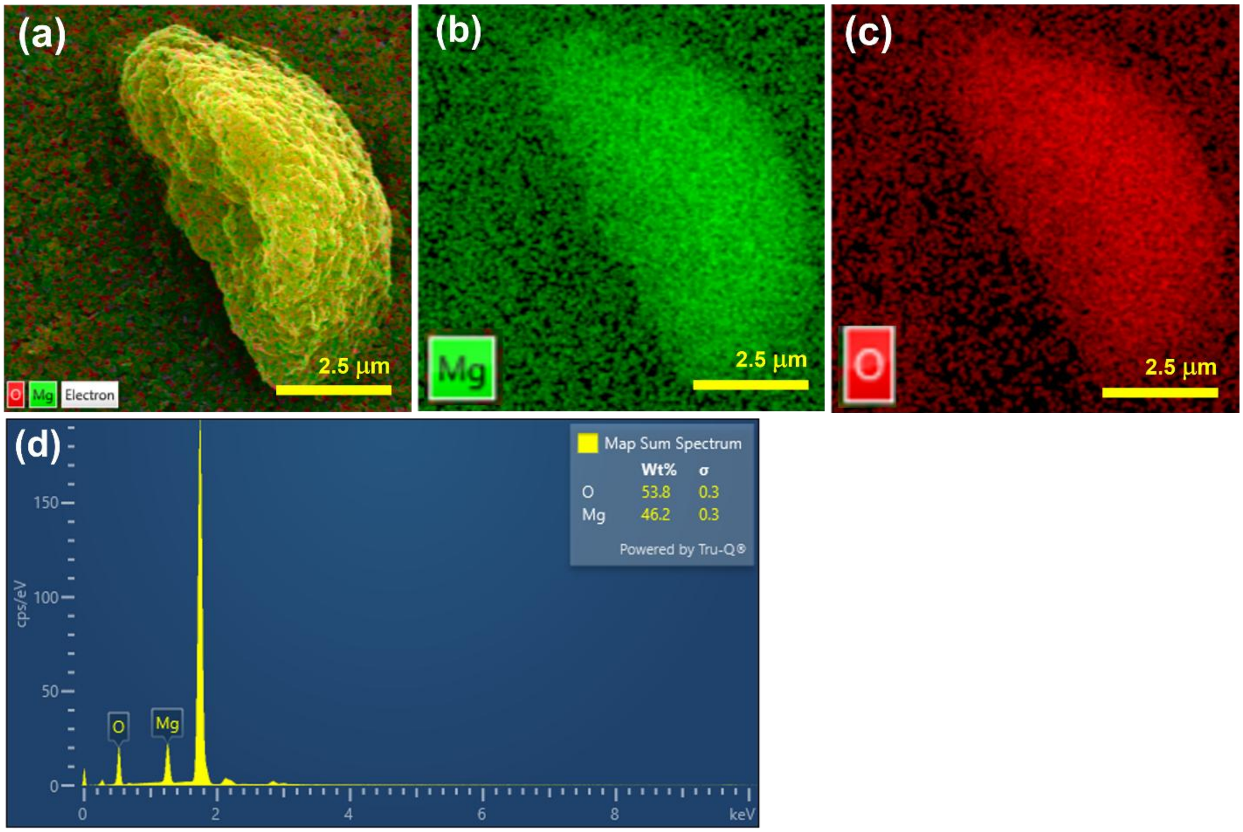

Figure S2 EDX image and its corresponding (d) individual elements in the $Mg(OH)_2$ NPs.

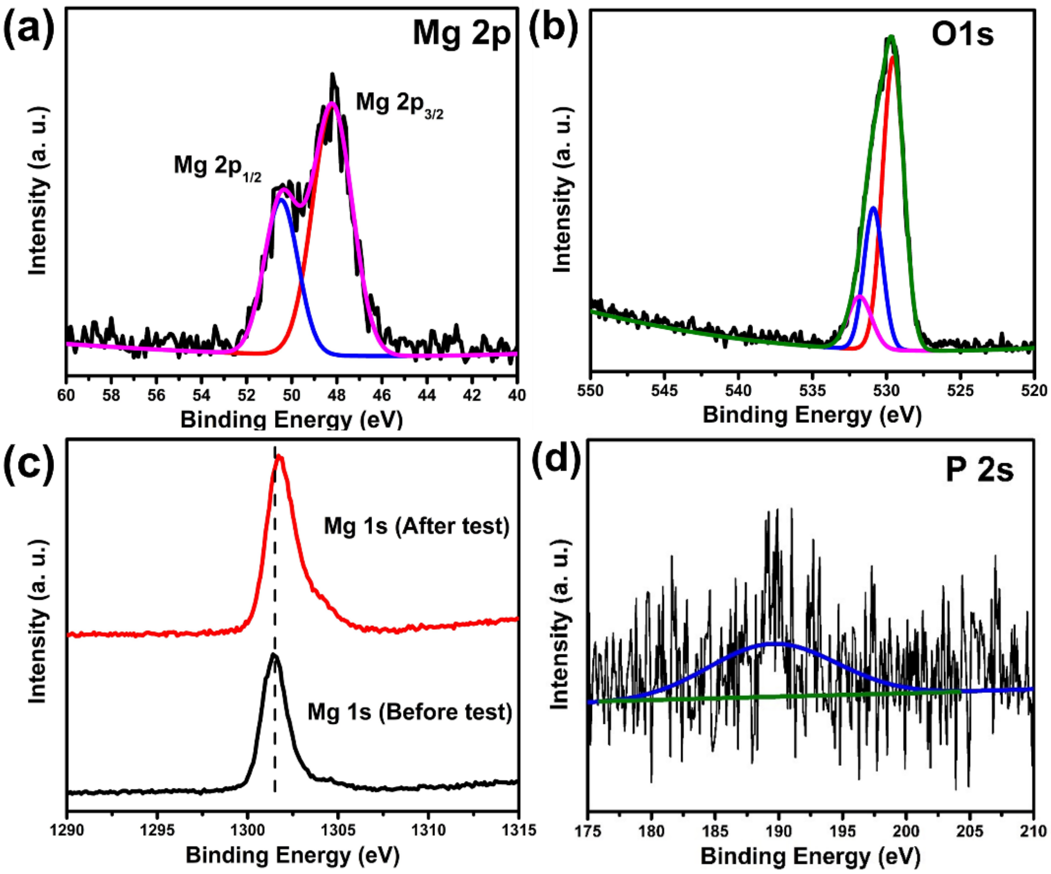

Figure S3: (a) Mg 2p spectrum and (b) O 1s spectrum before the adsorption test. (c) Comparison of Mg 1s before and after the adsorption test, and (d) P 2s spectrum after the adsorption test.

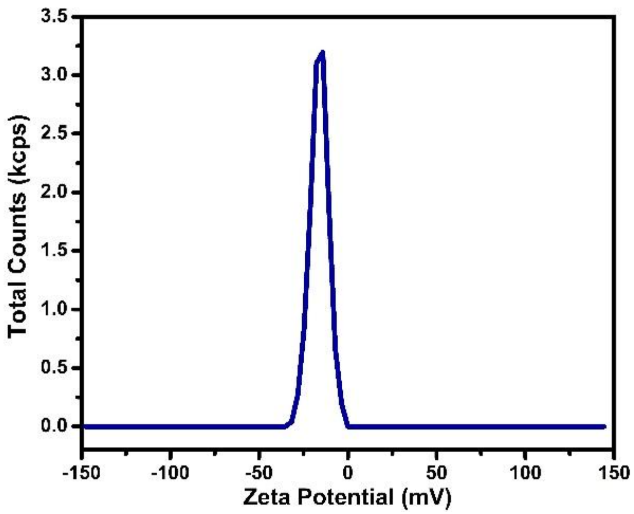

Figure S4: Spectrum from zeta potential analysis showing high counts of negative ions

Equation 1: Determination of the Freundlich isotherm of the phosphate ions adsorption.

Linearized Freundlich Equation (for plotting) is given by:

$$\log q_e = \log K_f + \frac{1}{n}\log C_e$$

where log C_e is plotted on the x-axis against log q_e on the y-axis giving a slope represented by 1/n and an intercept equal to log K_f

From the plot:

Slope (1/n) = 0.5077. Therefore n = 1.9697

Intercept (log K_f) = 0.6868. Therefore K_f = 4.8570

Equation 2: Determination of the Langmuir isotherm of the phosphate ions adsorption.

Linearized Langmuir Equation (for plotting) is given by:

$$\frac{1}{q_e} = \frac{1}{q_m K_L C_e} + \frac{1}{q_m} \quad \text{or} \quad \frac{C_e}{q_e} = \frac{1}{q_m K_L} + \frac{C_e}{q_m}$$

where $1/C_e$ is plotted on the x-axis against $1/q_e$ on the y-axis giving a slope represented by $1/q_m K_L$ and an intercept equal to $1/q_m$

From the plot:

Intercept ($1/q_m$) = 0.0242. Therefore q_m = 41.3223

Slope ($1/q_m K_L$) = 0.3811. Therefore, $q_m K_L = 2.62398$, and $K_L = 0.0635$

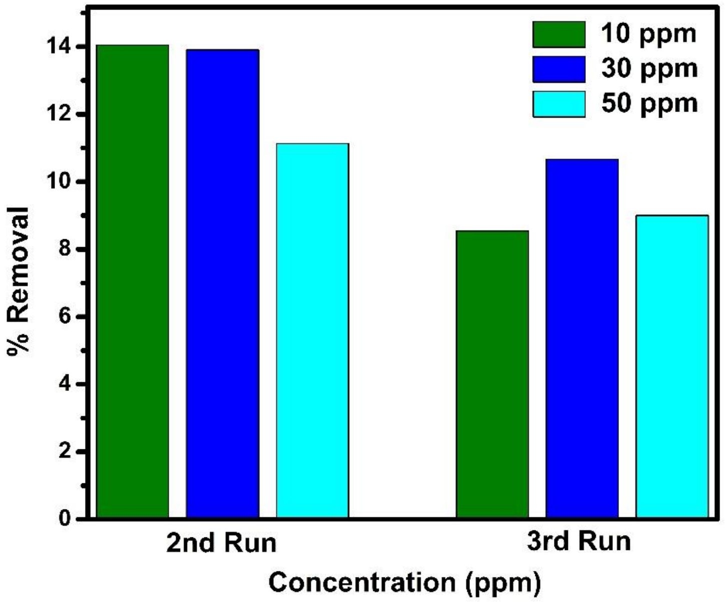

Figure S5: Phosphate removal results after 2nd and 3rd recycle tests using 10, 30, and 50 ppm phosphate solutions.

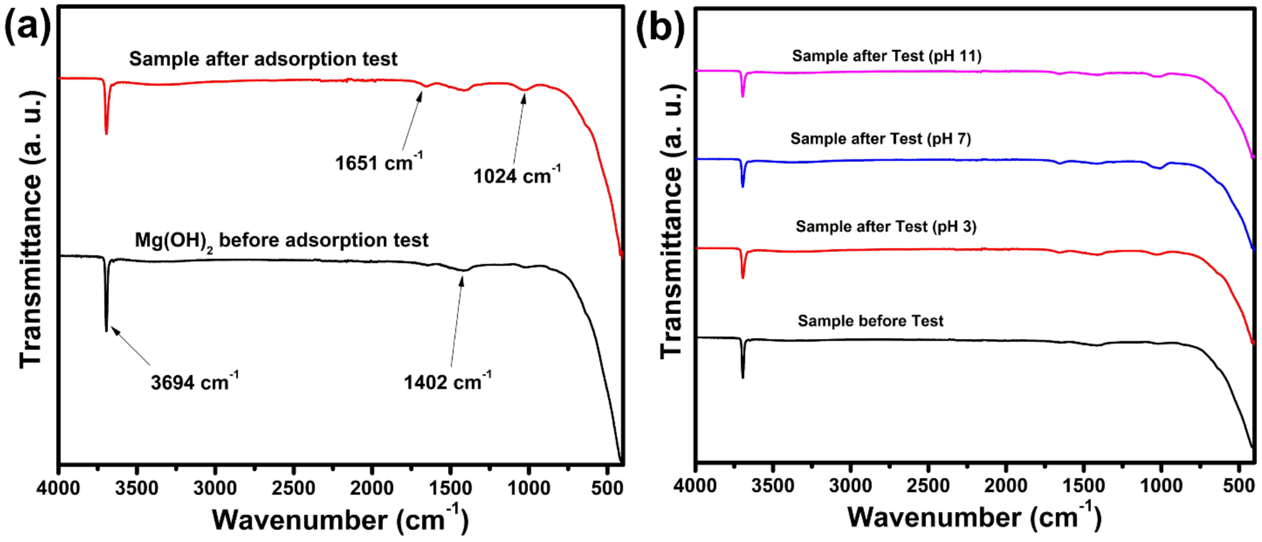

Figure S6: FTIR spectrum of samples before and after adsorption tests.

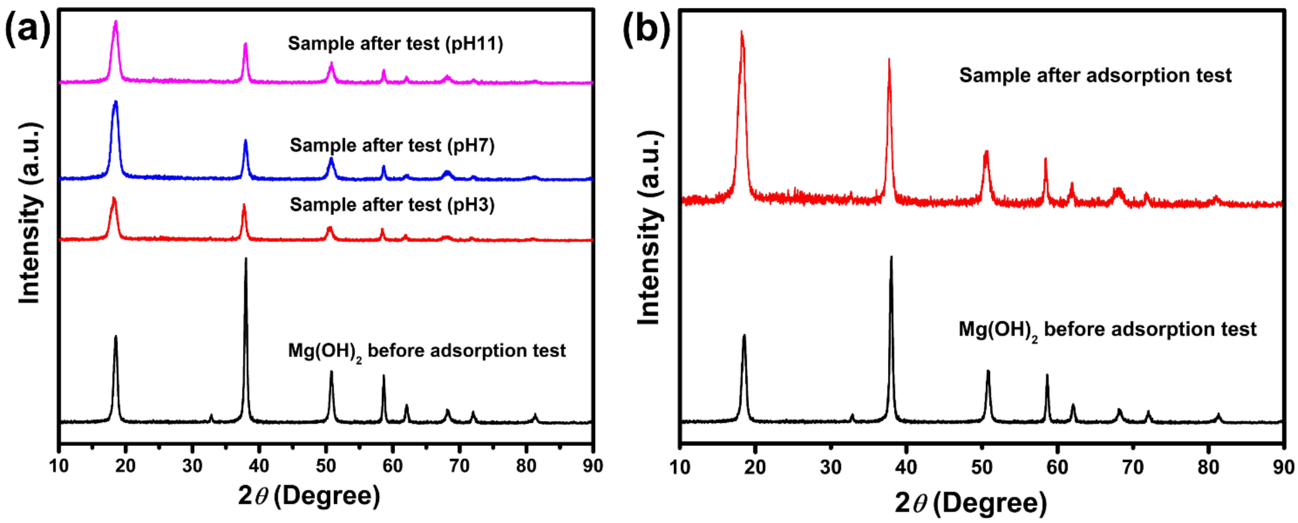

Figure S7: XRD spectrum of the samples before and after adsorption tests.
